# Deoxyshikonin Inhibits Influenza A Virus Infection at an Early Stage

**DOI:** 10.3390/ijms26178158

**Published:** 2025-08-22

**Authors:** Won-Kyung Cho, Jin Yeul Ma

**Affiliations:** Korean Medicine (KM) Application Center, Korea Institute of Oriental Medicine, 70 Cheomdan-ro, Dong-gu, Daegu 41062, Republic of Korea

**Keywords:** Deoxyshikonin (DS), influenza a virus (IAV), antiviral, Hemagglutinin (HA), virus-eradication effect

## Abstract

Deoxyshikonin (DS) is a derivative of shikonin, the main compound present in *Lithospermi radi*, the root of *Lithospermum erythrorhizon* Siebold and Zucc. In this study, we investigated the antiviral effects of DS using Influenza A/PR8/34, which expresses green fluorescent protein (GFP) as well as wild-type PR8/34 H1N1 Influenza A virus (IAV). Fluorescence microscopy and flow cytometry results showed that DS from 1.25 to 5 µM significantly and dose-dependently inhibited PR8-GFP IAV infection. A plaque assay confirmed the inhibitory effect of DS against H1N1 IAV infection. Consistently, immunofluorescence results showed that DS suppresses IAV protein expression. Time-of-drug-addition and hemagglutination inhibition assays revealed that DS exhibits anti-influenza virus efficacy by blocking the viral attachment and penetration into the cells and has a direct virus-eradication effect in the early stages of infection. However, DS did not repress neuraminidase activity. Our findings suggest that DS could be used not only to protect against the early stages of IAV infection, but also to treat influenza virus infections in combination with NA inhibitors.

## 1. Introduction

Every year, there are three to five million cases of pandemic influenza infections worldwide, resulting in 650,000 deaths from respiratory diseases associated with influenza viruses [1]. Influenza viruses, categorized as A, B, C, and D, belong to the Orthomyxoviridae family [2]. Among them, influenza A viruses (IAV) are the primary agents, causing epidemics and pandemics [1]. Influenza viruses have a negative-sense, single-stranded, segmented RNA genome. IAV contains the following RNA-dependent RNA polymerase complex, comprised of PA (polymerase acidic), PB1 (polymerase basic 1), and PB2 (polymerase basic 2), NS1 (nonstructural 1), NP (nucleoprotein), M1, M2 (matrix 1 and 2), HA (hemagglutinin), and NA (neuraminidase) proteins. Due to error-prone RNA-dependent replication, IAV generates antigenic shifts and point mutations in hemagglutinin and/or neuraminidase proteins [2]. Several antiviral agents against IAV infection have been developed to target IAV proteins, including M2, NA, and PA, and have been FDA-approved for clinical use. However, it has been reported that existing anti-IAV drugs have limitations, including side effects and drug-resistant strains [3,4]. M2 inhibitors such as amantadine and rimantadine are not effective on the influenza B virus and have been reported to induce common side effects, such as dizziness, insomnia, anxiety, headache, and gastrointestinal upset [5,6]. NA inhibitors, including oseltamivir, zanamivir, and peramivir, are analogs of the substrate for the active site of neuraminidase, thereby blocking the cleavage of hemagglutinin sialic acid and preventing the release of viral progeny [7]. Although NA inhibitors are still used to treat IAV-associated diseases, drug-resistant variants with various mutations in the active site of the NA gene have been discovered since the 2009 influenza pandemic [5,7,8] and reported to have side effects, including headache, nausea, vomiting, and coughing [5]. PA inhibitors such as baloxavir, which inhibit IAV replication by repressing RNA transcription, have been used to treat IAV infection. However, the high rate of IAV with mutations in the PA active site was detected during the 2018 to 2019 season [8], alongside side effects such as diarrhea, bronchitis, nausea, and sinusitis. Several other antiviral candidates, such as Favipiravir, pimodivir, enisamium, and flufirvitide 3, are undergoing clinical trials; however, the results have not been reported [8].

Deoxyshikonin (DS), known as shikon or Zicao, is found in several medicinal herbs, including *Lithospermi* radix (the root of *Lithospermum erythrorhizon* Siebold and Zucc.), *Arnebia decumbens*, and *Arnebia euchroma*. DS has been demonstrated to have various pharmacological effects, including anti-cancer [9,10,11,12], wound-healing [13], antibacterial [14], and antifungal effects [15]. Additionally, Li et al. have discovered that DS, one of the naphthouquinones in *Lithospermum erythrohizon*, has anti-tobacco mosaic virus (TMV) activity [16]. Recently, DS was reported to inhibit rotavirus replication by regulating autophagy and oxidative stress [17]. However, the anti-influenza virus effect of DS has not been reported until now. In the present study, we demonstrate that DS exhibits a potent inhibitory effect against IAV infection by preventing the virus from binding to and entering cells in the early stages of IAV infection.

## 2. Results

### 2.1. Effect of DS on Cell Viability

**Figure 1:**
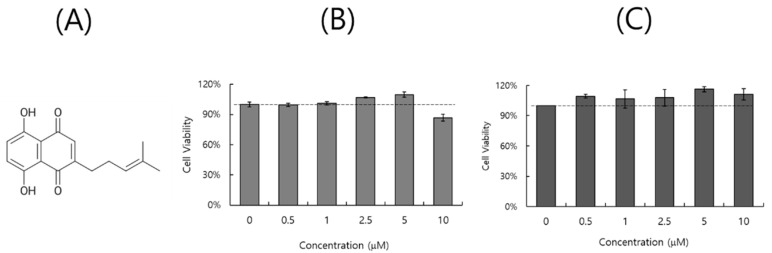
The structure (**A**) and toxicity determination of DS in A549 (**B**) and RAW 264.7 (**C**) cells. The cells were treated with DS at 0, 0.5, 1, 2.5, 5, or 10 µM and incubated for 24 h at 37 °C. CCK-8 reagents were added to each cell to examine the cytotoxicity of DS. The data represent the mean ± standard deviation, based on three independent triplicate experiments.

We used a CCK-8 assay to examine the toxicity of DS (Figure 1A) on A549 and RAW 264.7 cells. When cell viability was checked 24 h post-treatment with DS at the indicated concentrations (serially diluted from 10 µM), DS did not exhibit a significant toxic effect for up to 5 µM in A549 cells and 10 µM in RAW 264.7 cells (Figure 1B,C). The cytotoxicity experiments were performed in triplicate.

### 2.2. Anti-Influenza Viral Effect of DS

**Figure 2:**
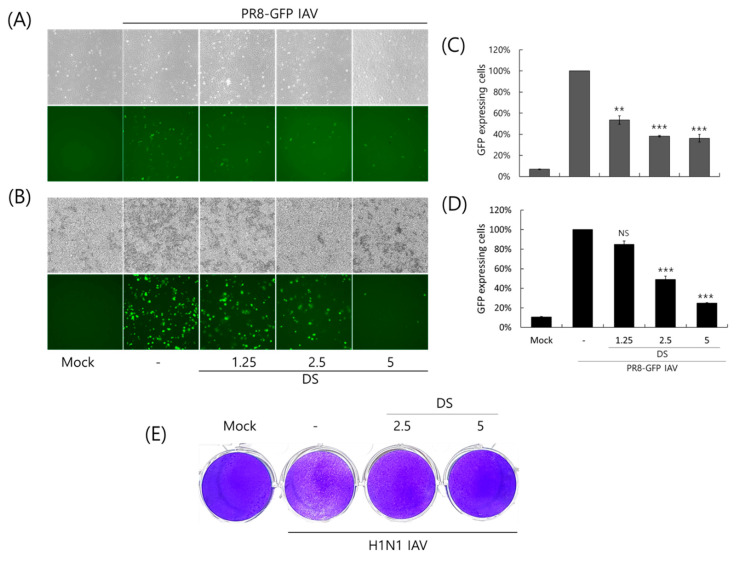
Antiviral effect of DS against H1N1 IAV infection. (**A**–**D**) DS at the indicated concentrations and PR8-GFP IAV (10 MOI) were co-incubated for 1 h at 4 °C. The mixtures were added to A549 (**A**,**C**) or RAW 264.7 (**B**,**D**) cells for 2 h at 37 °C. The cells were washed with PBS and further incubated for 24 h at 37 °C. (**A**,**B**) GFP expression in the cells infected by IAV was observed using a fluorescence microscope with 200× magnification. (**C**,**D**) The cells were fixed with paraformaldehyde, and the levels of GFP expression were detected using flow cytometry. (**E**) H1N1 IAV (10 MOI) and DS (2.5 or 5 µM) were mixed at 4 °C for 1 h and cotreated with MDCK cells at 37 °C for 1 h. After washing, the cells were overlaid with 1.5% agarose containing 1 µg/mL trypsin-treated TPCK and incubated for 72 h. The cells were fixed with paraformaldehyde and stained with 1% crystal violet. The data denote the mean ± standard deviation, based on triplicate experiments. Statistical significance was evaluated via Student’s unpaired *t*-test. *** *p* < 0.001 and ** *p* < 0.005 compared with PR8-GFP IAV-infected group; Mock, medium only; NS, no significance.

To investigate the antiviral effect of DS against the influenza A virus, we used the GFP-expressing Influenza A/PR8/34 virus (PR8-GFP IAV). PR8-GFP IAV in the presence of DS at the indicated concentrations was infected into A549 or RAW 264.7 cells and incubated for 24 h at 37 °C to express GFP. As shown in Figure 2A,B, A549 and RAW 264,7 cells infected by PR8-GFP IAV expressed green fluorescent proteins. However, in the presence of DS, the levels of GFP expression were significantly decreased in a concentration-dependent manner. We confirmed the inhibitory effect of DS on the expression of GFP via PR8-IAV using FACS analysis with the cells infected by PR8-GFP IAV in the presence of DS at the indicated concentrations. When the GFP expression levels in each group were compared with the PR8-GFP IAV control group and calculated as the relative intensity, the DS strongly inhibited viral GFP expression in both cells (Figure 2C,D), which is consistent with Figure 2A,B. Next, we further affirmed the anti-influenza A viral impact of DS using a plaque inhibition assay. To perform the plaque assay, H1N1 IAV in the presence of 0, 2.5, or 5 μM DS was infected into MDCK cells and incubated for 3 days. Figure 2E presents an example of DS that significantly blocked plaque formation by the H1N1 IAV infection. These results suggest that DS has antiviral efficacy against influenza A virus infection. All antiviral experiments were conducted in triplicate.

### 2.3. Repressive Effect of DS on IAV Protein Expression

**Figure 3:**
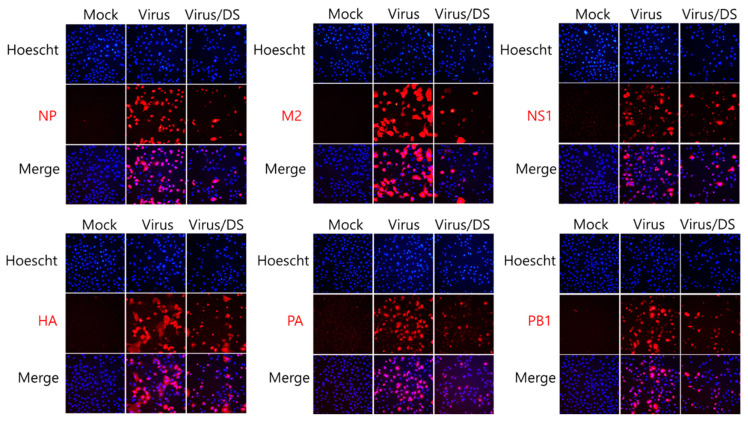
Inhibitory effects of DS on influenza virus protein expression. A549 cells were co-infected with 10 MOI H1N1 IAV and 5 µM DS for 24 h. The cells were then fixed and stained red with antibodies against IAV proteins. The nuclei of the cells were stained blue with Hoechst 33342. The viral proteins in red and nuclei in blue were colocalized and visualized using a fluorescence microscope at a magnification of 200×. Mock, medium only.

Because DS exerted strong inhibitory efficacy against IAV infection, as shown in Figure 2, we next investigated whether DS influences the IAV protein expression using immunofluorescence analysis. H1N1 IAV, in the presence and absence of DS, was infected in A549. At 24 h post-infection, the cells were fixed with paraformaldehyde. The expressions of IAV proteins, including NP, M2, NS1, HA, PA, and PB1, were detected using red color Alexa 594-tagged antibodies. The nuclei were stained in blue with Hoechst 33342. Figure 3 shows that DS potently represses all IAV proteins, consistent with the results of Figure 2.

### 2.4. Inhibitory Effect of DS on IAV Infection at an Early Stage

**Figure 4:**
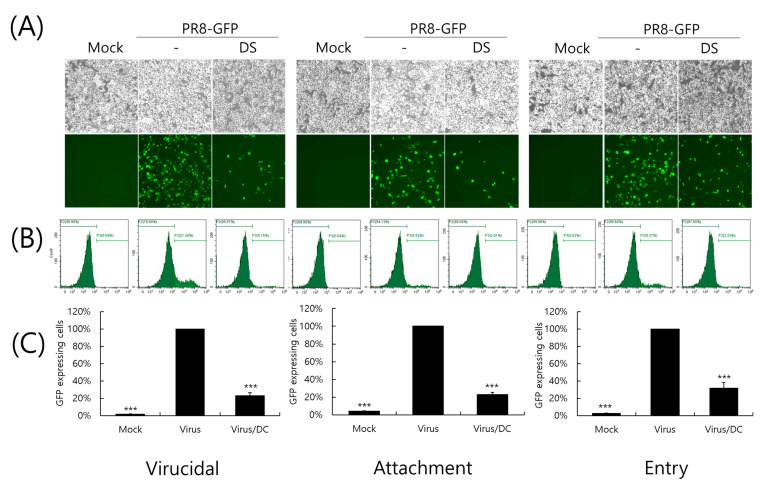
The effect of DS on early stages of IAV infection. The effect of DS on viral binding, penetration, and virucidal ability was examined using a time-of-drug-addition experiment with different incubation temperatures and time conditions for the infection of cells with PR8-GFP IAV and DS, as described in the Materials and Methods section. (**A**) GFP-expressing cell images were captured using fluorescence microscopy at a magnification of 200×. (**B**,**C**) The cells fixed with paraformaldehyde were analyzed using FACS to count the number of GFP-expressing cells in each group. The data denote the mean ± standard deviation based on triplicate experiments. Statistical significance was evaluated via Student’s unpaired *t*-test. *** *p* < 0.001 compared with the PR8-GFP IAV-infected group. Mock, medium only.

Next, we conducted a time-of-addition assay to investigate whether DS could affect the influenza virus during the early stages of infection. First of all, when we examined whether DS has a direct killing effect before it binds to or enters into the cells, we found that DS has a potent virucidal effect, as shown in the left panel of Figure 4. When we further checked the effect of DS on viral binding and penetration into the cells, DS significantly inhibited both viral binding and entry into the cells. These results indicate that DS prevents IAV infection at an early stage.

### 2.5. Effect of DS on Hemagglutination by IAV Infection

**Figure 5:**
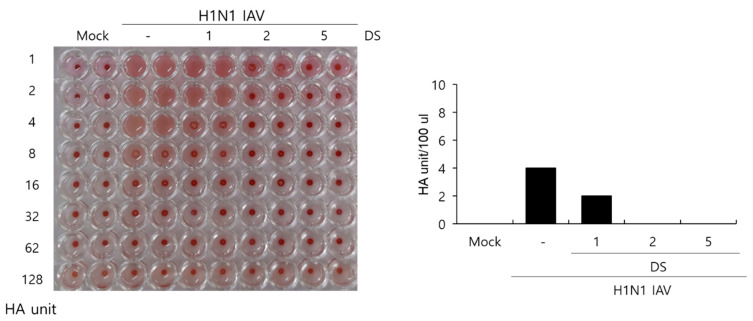
DS reduced hemagglutination caused by IAV infection. H1N1 IAV and DS (0, 1, 2, or 5 µM) were mixed for 1 h at 4 °C, and the mixture was added to cells for 24 h. Each supernatant was harvested, serially diluted, and incubated with 1% RBC cells. Mock, medium only.

Hemagglutinin is a protein that is important for IAV to bind to and enter cells, and it induces the hemagglutination of red blood cells. Because DS exhibited a strong suppressive effect on IAV binding and penetration into the cells, as presented in Figure 4, we next examined whether DS influences hemagglutination by IAV HA proteins using chicken red blood cells. Figure 5 shows that the H1N1 IAV-infected control in the absence of DS causes agglutination of the RBC, whereas DS inhibits hemagglutination from 1 µM. The H1N1 IAV-infected control showed 4 HA units. However, HA units in the presence of 1 µM DS were two-fold lower than those in the IAV-infected control. In particular, 2 and 5 µM of DS completely prevented hemagglutination caused by IAV infection. These results indicate that DS inhibits the HA protein, blocking IAV attachment and penetration into the cells.

### 2.6. Effect of DS on IAV Neuraminidase Activity

**Figure 6:**
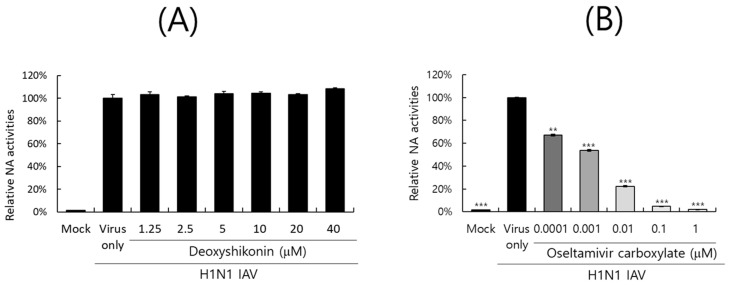
Effect of DS on IAV neuraminidase activity. DS (**A**) or oseltamivir carboxylate (**B**), serially diluted, was mixed with H1N1 IAV in a black 96-well plate. The neuraminidase inhibition assay was conducted according to the manufacturer’s instructions. NA activities were measured as fluorescence values using excitation at 365 nm and emissions at 445 nm. The data denote the mean ± standard deviation based on triplicate experiments. Statistical significance was evaluated using Student’s unpaired *t*-test. *** *p* < 0.001 and ** *p* < 0.005 compared with H1N1 IAV-infected group. Mock, medium only.

The neuraminidase of IAV facilitates the release of progeny virions from infected cells and is one of the main targets of antiviral drugs such as oseltamivir or zanamivir. We next explored whether DS could affect the NA activity of IAV. DS or oseltamivir carboxylate, a positive control, was serially diluted and mixed with H1N1 IAV according to the NA-Fluor Influenza Neuraminidase assay kit. After substrate addition, the activity was determined by fluorescence differences. As shown in Figure 6A, DS did not exhibit a neuraminidase inhibitory effect. Oseltamivir carboxylate dose-dependently blocked the NA activity of H1N1 IAV (Figure 6B). These results imply that DS does not affect NA activity and thus does not inhibit the release of viral progeny from infected cells during the late stages of infection.

## 3. Discussion

Influenza viruses are infectious agents that cause enormous damage worldwide. Numerous attempts have been made to prevent and treat influenza viral infections, but no perfect antiviral drug has been developed to date. In this study, we examined the effect of DS against influenza virus infections and its potential as an antiviral agent. When we investigated the effect of DS against influenza viruses using GFP-expressing influenza viruses, we found that DS significantly and dose-dependently repressed GFP expression by IAV infection in A549 and RAW 264.7 cells (Figure 2A–D). Consistently, DS treatment reduced plaque formation caused by H1N1 IAV infection (Figure 2E). DS significantly decreased the expression of the influenza virus proteins M2, NP, HA, NS1, PA, and PB1. To examine the strong antiviral effect of DC against IAV infection, which is associated with the inhibition of IAV infection at an early stage, we performed a time-of-drug-addition assay using different infection conditions. We found that DS exerts a strong inhibitory effect on IAV attachment to and penetration into cells (Figure 4). DS prevented IAV attachment to and penetration into cells by 80% compared to the virus-infected control. Because hemagglutinin of the influenza virus binds to sialic acid-linked glycoprotein receptors on target cells during early infection [18], there have been trials conducted to block viral infection by modulating the interaction between receptors and HA. Several studies suggest that the triterpene [19] and pentacyclic triterpene structures [20] could prevent viral binding and inhibit HA, thereby protecting the cells from IAV infection. Chang YJ et al. demonstrated computer modeling to screen an anti-IAV candidate that inhibits the interaction between sialic acid and HA [21]. Other clinical trials targeting HA have been conducted, including small molecules [22,23,24,25] that inhibit HA, such as arbidol [26] and flupirubitide 3 [27], neutralizing antibodies [28] to conserved epitopes of HA, and the sialidase fusion protein DAS181 [29,30], but the results have not yet been reported [8]. In previous research, aureonitol [31], amentoflavone [32], neoechinulin B [33], ginsenoside Rk1 [34], and isoquercitrin [35] in natural plants have been shown to prevent IAV infection by inhibiting IAV binding and entry through the modulation of HA at an early stage of infection. A hemagglutination inhibition assay using RBC confirmed that DS suppresses hemagglutination caused by IAV infection (Figure 5). These results indicate that DS prevents IAV attachment to the cell by blocking the HA of IAV. Additionally, DS eradicated the influenza A virus before it binds to the cells. NA is the primary target of antiviral drugs currently in use, such as oseltamivir, zanamivir, and peramivir. However, our findings indicate that DS does not inhibit NA (Figure 6). Although DS did not repress the NA activity of IAV, it did prevent IAV infection by interfering with hemagglutinin and inducing virucidal action during the early points of infection. These findings suggest that DS may have the potential to be used as an anti-IAV agent in combination with NA inhibitors, which target the later stages of infection.

## 4. Materials and Methods

### 4.1. Reagents, Cell Culture, and Viruses

Deoxyshikonin (PubChem CID: 98914) was used with the screen-well natural product library BML2865 in Enzo Life Sciences (Farmingdale, NY, USA) and Sigma-Aldrich product (CAS number 43043-74-9, 95% purity) (St. Louis, MO, USA). A549(ATCC CCL-185), human lung adenocarcinoma cells, and RAW 264.7 (ATCC TIB-71), mouse leukemia monocyte macrophage cells, were maintained in a Roswell Park Memorial Institute medium (Hyclone, Logan, UT, USA) with 10% fetal bovine serum and 100 U/mL penicillin and streptomycin_._ MDCK (ATCC CCL-34), Madin–Darby canine kidney cells, were cultured in Dulbecco’s Modified Eagle Medium containing 10% fetal bovine serum and 100 U/mL penicillin and streptomycin. All cells were maintained at 37 °C in the presence of 5% CO_2._ Green fluorescent protein (GFP)-expressing Influenza A/PR8/34 (PR8-GFP) and A/PR8/34 (H1N1) viruses were kindly gifted from Prof. Jong-Soo Lee (Chungnam National University, Daejeon, Republic of Korea). The HBPV-VR-32 (H3N2) influenza A virus was obtained from the Korea Bank for Pathogenic Viruses (KBPV, Seoul, Republic of Korea). All viruses were propagated in 10-day-old chicken embryos and stored at −70 °C. All virus-related experiments were performed at Biosafety Level 2.

### 4.2. Cytotoxicity Determination

RAW 264.7 cells at a density of 1 × 10^5^ cells/well and A549 cells at a density of 5 × 10^4^ cells/well were seeded and grown in 96 wells for 24 h. DS at 0.5, 1, 2.5, 5, or 10 µM was added to the cells and incubated for 24 h. The CCK-8 reagent (Dojindo, Rockville, MD, USA) was added to the cells for 2 h, and then absorbance at 450 nm was detected using a GloMax ELISA microplate reader (Promega, Madison, WI, USA).

### 4.3. Antiviral Assay Against Influenza Virus Infection

DS at the indicated concentration and 10 MOI Influenza A/PR8-GFP virus (PR8-GFP IAV) were co-incubated for 1 h at 4 °C. A549 or RAW 264.7 cells were treated with the mixtures for 2 h at 37 °C. The cells were washed with PBS and further incubated for 24 h. GFP expression levels were detected using fluorescent microscopy and fluorescence-activated cell sorting (FACS) analysis. For FACS analysis, the cells were fixed with 4% paraformaldehyde and analyzed using the CytoPLEX flow cell counter (Beckman Coulter Inc., Pasadena, CA, USA). For a plaque reduction assay, H1N1 IAV was preincubated with DS (2.5 or 5 µM) at 4 °C for 1 h, and then added into MDCK cells for 2 h at 37 °C. After washing with PBS, the cells were overlaid with DMEM containing 1.5% agar and 1 µg/mL trypsin-treated TPCK. After incubation for 72 h, the cells were fixed with 4% paraformaldehyde and stained with 1% crystal violet after removal of the agar overlay.

### 4.4. Viral Attachment, Entry, and Virucidal Assay

To investigate the effect of DS on the influenza viral attachment, entry, or virucidal stages, we used different incubation conditions for the cotreatment of DS and IAV. For attachment stages, PR8-GFP IAV (10 MOI) and DS (5 µM) were incubated with RAW 264.7 cells for 30 min at 4 °C. After washing with PBS to remove the unbound virus and DS, the cells were further incubated for 24 h at 37 °C. For the entry stage, PR8-GFP IAV was added to the cells for 30 min at 4 °C. After washing the cells with PBS, DS was added to the cells for 30 min at 37 °C. The cells were further incubated for 24 h at 37 °C after removal of the virus and DS. To check the virucidal effect of DS, PR8-GFP IAV and DS were co-incubated for 30 min at 4 °C. The virus and DS mixture were added to the cells for 30 min at 37 °C. After removal of DS and the virus with PBS washing, the cells were further incubated for 24 h at 37 °C. The GFP expression via IAV infection was detected using fluorescent microscopy at 200× magnification and flow cytometry.

### 4.5. Immunofluorescence Analysis Against IAV Proteins

H1N1 IAV at an MOI of 10 and 5 µM DS were incubated for 1 h at 4 °C. The mixture was cotreated with A549 cells. At 24 h post-infection, the cells were fixed with 4% paraformaldehyde for 10 min. After blocking with 1% BSA-containing PBS, the cells were incubated with NP, NS1, M2, HA, PA, and PB1 antibodies (GeneTex, Irvine, CA, USA) for 12 h at 4 °C. The cells were washed with 0.05% Tween 20-containing PBS and incubated with an Alexa Fluor 594-conjugated secondary antibody (Thermo Fisher Scientific, Waltham, MA, USA) for 1 h at 37 °C in the dark. The nuclei were stained with Hoechst 33342 (Thermo Fisher Scientific, Waltham, MA, USA). The images of viral proteins in red and nuclei in blue were detected using fluorescent microscopy.

### 4.6. Hemagglutination Inhibition Assay Using RBC

DS was serially diluted and mixed with H1N1 IAV for 1 h at 4 °C. RAW 264.7 cells infected with the mixtures were incubated for 24 h at 37 °C. The supernatant of each cell was serially diluted and mixed with chicken red blood cells (Innovative Research, Inc., Southfield, MI, USA) in a U-bottom 96-well plate for 1 h. RBCs in the virus-infected well were hemagglutinated. Hemagglutination inhibition was expressed as HA titers in comparison with the virus control.

### 4.7. Neuraminidase Inhibition Assay

A neuraminidase inhibition assay was conducted according to the NA-Fluor influenza Neuraminidase Assay Kit (Life Technologies, Carlsbad, CA, USA). DS was serially diluted from 40 μM to 1.25 μM, and oseltamivir carboxylate (Aobious Inc., MA, USA), a specific neuraminidase inhibitor, was serially diluted from 1 μM to 0.0001 μM. H1N1 IAV was added to each sample, mixed and incubated for 30 min, followed by the addition of the NA-Fluor substrate for 1 h at 37 °C. After stopping the reaction, neuraminidase activity was detected with a fluorescence spectrometer (Promega, Madison, WI, USA) with excitation at 365 nm and emission at 445 nm.

### 4.8. Statistical Analysis

The data were presented as the mean ± standard deviation based on three independent experiments. Statistical significance was assessed via Student’s unpaired *t*-test. Statistical significance was defined as *** *p* < 0.001 and ** *p* < 0.005 compared with the IAV-infected group.

## 5. Conclusions

DS protects the cell from influenza A virus infection by inhibiting the binding and entry of the virus. Notably, DS eradicated the influenza virus before the virus entered the cells. Although further study is needed to confirm the anti-IAV effect of DS using an IAV-infected animal model or lung organoid, our findings suggest that DS could be used in combination with NA inhibitors to treat influenza virus infection, as well as to attenuate the early stages of IAV infection.

## Data Availability

All data are included in the article.
